# Ontology-based systematic representation and analysis of traditional Chinese drugs against rheumatism

**DOI:** 10.1186/s12918-017-0510-5

**Published:** 2017-12-21

**Authors:** Qingping Liu, Jiahao Wang, Yan Zhu, Yongqun He

**Affiliations:** 1 Guangzhou University of Chinese Medicine, Guangzhou, Guangdong China; 2 Unit for Laboratory Animal Medicine, Department of Microbiology and Immunology, Center for Computational Medicine and Bioinformatics, and Comprehensive Cancer Center, University of Michigan Medical School, 1301 MSRB III, 1150 W. Medical Dr, Ann Arbor, MI 48109 USA; 3 Institute of Information on Traditional Chinese Medicine, China Academy of Chinese Medical Sciences, Beijing, 100700 China

**Keywords:** Traditional Chinese medicine, Rheumatism, Ontology, OCMR, Bioinformatics

## Abstract

**Background:**

Rheumatism represents any disease condition marked with inflammation and pain in the joints, muscles, or connective tissues. Many traditional Chinese drugs have been used for a long time to treat rheumatism. However, a comprehensive information source for these drugs is still missing, and their anti-rheumatism mechanisms remain unclear. An ontology for anti-rheumatism traditional Chinese drugs would strongly support the representation, analysis, and understanding of these drugs.

**Results:**

In this study, we first systematically collected reported information about 26 traditional Chinese decoction pieces drugs, including their chemical ingredients and adverse events (AEs). By mostly reusing terms from existing ontologies (e.g., TCMDPO for traditional Chinese medicines, NCBITaxon for taxonomy, ChEBI for chemical elements, and OAE for adverse events) and making semantic axioms linking different entities, we developed the Ontology of Chinese Medicine for Rheumatism (OCMR) that includes over 3000 class terms. Our OCMR analysis found that these 26 traditional Chinese decoction pieces are made from anatomic entities (e.g., root and stem) from 3 *Bilateria* animals and 23 *Mesangiospermae* plants. Anti-inflammatory and antineoplastic roles are important for anti-rheumatism drugs. Using the total of 555 unique ChEBI chemical entities identified from these drugs, our ChEBI-based classification analysis identified 18 anti-inflammatory, 33 antineoplastic chemicals, and 9 chemicals (including 3 diterpenoids and 3 triterpenoids) having both anti-inflammatory and antineoplastic roles. Furthermore, our study detected 22 diterpenoids and 23 triterpenoids, including 16 pentacyclic triterpenoids that are likely bioactive against rheumatism. Six drugs were found to be associated with 184 unique AEs, including three AEs (i.e., dizziness, nausea and vomiting, and anorexia) each associated with 5 drugs. Several chemical entities are classified as neurotoxins (e.g., diethyl phthalate) and allergens (e.g., eugenol), which may explain the formation of some TCD AEs. The OCMR could be efficiently queried for useful information using SPARQL scripts.

**Conclusions:**

The OCMR ontology was developed to systematically represent 26 traditional anti-rheumatism Chinese drugs and their related information. The OCMR analysis identified possible anti-rheumatism and AE mechanisms of these drugs. Our novel ontology-based approach can also be applied to systematic representation and analysis of other traditional Chinese drugs.

**Electronic supplementary material:**

The online version of this article (10.1186/s12918-017-0510-5) contains supplementary material, which is available to authorized users.

## Background

Rheumatic diseases are among the oldest diseases recognized. In the fourth century B.C., such diseases were first recognized by Hippocrates. In about the first century A.D., the term “rheuma” was first introduced to represent a flow of pain through joints of the body [1]. The word “rheumatism” appeared about 300 or 400 years ago, and subsumed both articular and non-articular musculoskeletal conditions. In 1802, William Heberden described the rheumatism as a common name for many aches and pains owing to very different causes [2]. More than 100 different conditions could be labelled as rheumatic diseases, including rheumatoid arthritis, osteoarthritis, systemic lupus erythematosus, scleroderma, osteoporosis, back pain, gout, fibromyalgia and tendonitis [3]. Rheumatoid arthritis, one of the most common rheumatic diseases, is characterized by persistent synovitis, systemic inflammation, and autoantibodies. Approximately 1% of the human population in the world is afflicted by rheumatoid arthritis [4]. Existing drugs for treating rheumatism include NSAID (non-steroidal anti-inflammatory drug) and steroids for controlling symptoms, and disease-modifying anti-rheumatic drugs (DMARDs) (e.g., methotrexate and leflunomide) for inhibiting the underlying immune process and long-term damages [4–6]. However, these drugs are in general far from satisfactory in terms of effectiveness and safety.

From over a thousand years of experience, many traditional Chinese drugs (TCDs), most of them made from Chinese herbs, have been found to be effective in treating rheumatic diseases. For example, *Tripterygium wilfordii* Hook F (TwHF), also called Common Threewingnut Root, Lei Gong Teng and Thunder God Vine, is a Chinese traditional remedy used to treat many rheumatic diseases such as rheumatoid arthritis, systemic lupus erythematosus, and psoriasis [7]. TwHF extract inhibits the production of cytokines and other mediators from mononuclear phagocytes by blocking the upregulation of a number of proinflammatory genes, including TNFα, COX2, interferon-γ, IL-2, prostaglandin, and iNOS [8, 9]. The therapeutic and adverse effects of the preparation are thought to be due to triptolide and tripdiolide, two diterpenoid compounds with epoxide groups and have immunosuppressive and anti-inflammatory effects of similar efficacy in vivo and in vitro [10–12]. The root of *Tripterygium hypoglaucum*, also called Tripterygii Hypoglauci Radix TCD, contains similar chemical compositions and have similar clinical efficacy compared with TwHF [13]. Other herbs, such as *Stephania tetrandra,* have proved to be effective in treating rheumatic diseases [14].

1Although the anti-rheumatism Chinese drugs have significant therapeutic effects, many of them induce adverse events (possible side effects). For example, Common Threewingnut Root TCD, extracted from TwHF, has proved to cause diarrhea, hair loss and nausea in some patients. Even the low dose (180 mg) of ethanol/ethyl acetate extract of TwHF can cause 1–3 loose stools per day, which lasts for 1–3 days [9]. Tripterygii Hypoglauci Radix TCD can also cause some common adverse events (AEs), such as nausea, disturbances, drowsiness, and paresthesia [15]. Both Tripterygii Hypoglauci Radix TCD and Common Threewingnut Root TCD are associated with reproductive system AEs such as necrospermia and hypomenorrhea [16]. *Aconitum carmichaelii* is commonly used in clinics for rheumatic diseases such as rheumatic fever and painful joints [17]. However, inadequate boiling time of *A. carmichaelii* Debeaux can cause aconitine poisoning [18].

The anti-rheumatism and AE mechanisms of traditional Chinese drugs (TCDs) still remain unclear. Overall, existing western medicine drugs (e.g., non-steroidal anti-TNF drugs and COX-2 inhibitors) have simple active ingredients and function through anti-inflammatory and immune suppressive mechanisms [19–21]. However, each TCD typically contains a number of chemical elements which may interact with each other. The functions of these different chemical elements and their interactions are largely unknown. For example, many anti-rheumatism TCDs, such as Common Threewingnut Root TCD [16], have also been used to treat cancer. Studies have also associated rheumatism with neoplastia and cancer [22–24]. However, it is still unclear what anticancer chemical elements exist in anti-rheumatism TCDs and how they possibly contribute to the combined anti-rheumatism effects and AEs.

In the big data and IT era, a biomedical ontology is a set of human- and computer-interpretable terms and relations that represent the entities in a specific biomedical domain and how these entities are related. Ontologies have played a critical role in biomedical data standardization, sharing, and analysis [25–28]. Many ontologies can be used to study TCDs. For example, the Institute of Information on Traditional Chinese Medicine in China has recently developed the TCM Decoction Pieces Ontology (TCMDPO; https://github.com/TCMOntology/TCMDPO), which can be used to represent TCDs. The NCBITaxon is an organism taxonomy ontology based on the NCBI Taxonomy system [29]. Plant Ontology (PO) describes plant anatomy, morphology and the stages of plant development [30]. The integrative cross-species anatomy ontology UBERON covers anatomical structures in animals [31]. The Chemical Entities of Biological Interest (ChEBI) ontologically represents and classifies over 50,000 molecular entities, esp. small chemical compounds, which are either products of nature or synthetic products used to intervene in the processes of living organisms [32]. The relationships among these chemical entities, their parent terms, and biological roles are also defined in ChEBI. The Ontology of Adverse Events (OAE) is a community-based biomedical ontology that represents various AEs after medical interventions (e.g., drug administration) [33]. These ontologies can be reused and integrated to represent and study anti-rheumatism TCDs.

In this study, we report our research of systematic information collection and development a community-driven Ontology of CM for Rheumatism (OCMR) that ontologically represents 26 anti-rheumatism Chinese drugs, their chemical ingredients, AEs, and related information. OCMR is developed by reusing many terms from existing ontologies (including those mentioned above) and using the state-of-the-art ontology engineering technologies [25, 27, 28, 34]. A systematical analysis of the OCMR-represented information allows us to generate new insights about these Chinese drugs.

## Methods

### Identification of anti-rheumatism TCDs and their related information

The identification of 26 anti-rheumatism TCDs was based on our own knowledge and from references, and all our annotated results were recorded in an Excel file (Additional file 1). For each TCD, the information we manually annotated included organism, anatomic entity, chemical ingredients, and AEs. The information of chemical ingredients for each TCD was extracted from reliable resources, mostly from peer-reviewed journal articles. The references for extracting the chemical ingredients are provided in the Additional file 1. We then mapped our identified information to corresponding ontologies. Specifically, TCDs were mapped to the TCM Decoction Pieces Ontology (TCMDPO; https://github.com/TCMOntology/TCMDPO), organisms mapped to NCBITaxon [29], animal anatomical entities to UBERON [31], plant anatomical entities to PO [30], chemicals to ChEBI [32], and AEs to OAE [33]. For those chemicals without ChEBI IDs, we submitted requests to ChEBI website (https://www.ebi.ac.uk/chebi/) to obtain new ChEBI IDs.For those AE terms not found in OAE, we added new OAE terms based on the standard OAE development procedure [33].

### OCMR ontology generation

The OCMR ontology design follows the Open Biomedical Ontologies (OBO) Foundry principles (e.g., openness and collaboration) [34]. Protégé 5.0 OWL ontology editor (http://protege.stanford.edu/) was used for ontology editing. OWL represents the format of W3C standard Web Ontology Language (OWL2) (https://www.w3.org/TR/owl-guide/). Ontofox (http://ontofox.hegroup.org/) [35] was used to extract subsets of related terms from different ontologies. In addition, we used Ontorat (http://ontorat.hegroup.org/) [36] to quickly add the annotations and relations between different entities.

### OCMR access, visualization, and licensing

The OCMR project website is located at GitHub:https://github.com/biomedontology/ocmr/. OCMR was also deposited in Ontobee (http://www.ontobee.org/ontology/OCMR) and NCBO BioPortal (http://bioportal.bioontology.org/ontologies/OCMR). The OCMR source code is freely available under the Creative Commons 3.0 License (http://creativecommons.org/licenses/by/3.0/), which allows OCMR users to freely distribute and use OCMR.

### OCMR ontology SPARQL query and knowledge analysis

The Ontobee SPARQL query web page (http://www.ontobee.org/sparql) was used to query OCMR to address specific questions. The knowledge stored in the OCMR ontology was also analyzed under the Protégé OWL editor and using Tableau (https://www.tableau.com/).

## Results

### Collection of data related to 26 anti-rheumatism traditional Chinese drugs (TCDs)

**Fig. 1:**
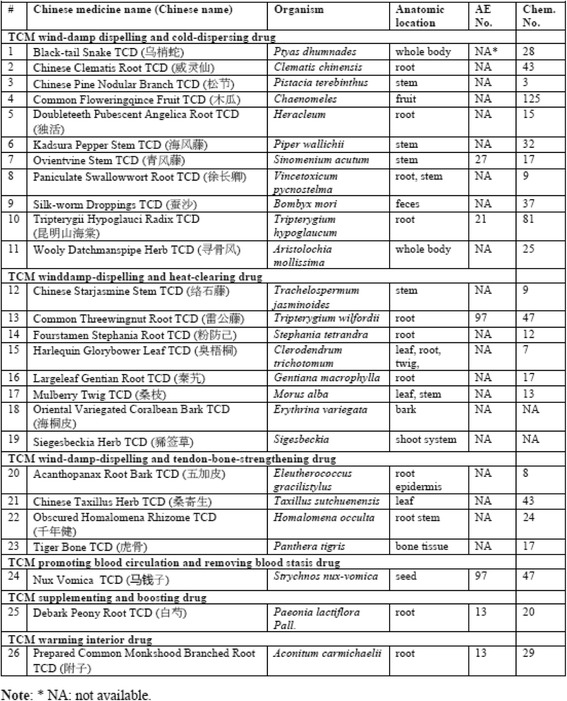
26 anti-rheumatism traditional Chinese drugs studied in this project

In total, we identified 26 anti-rheumatism TCDs (Fig. 1 and Additional file 1). These TCDs are made from anatomic parts (mostly from root or stem) of 26 different animal and plant organisms. Three TCDs were from animals, i.e., tiger bone, black-tail snake, and silk-worm droppings. The others were extracted from different plants (Fig. 1). For each TCD, we manually identified chemical ingredients and AEs reported from peer-reviewed articles (Additional file 1). These resources then became the basic information of our later ontology modeling, representation, and systematic analyses.

Our study identified 875 unique chemical elements in these 24 of these 26 TCDs. In our study, no chemical elements were found to be associated with Oriental Variegated Coralbean Bark and Siegesbeckia Herb Chinese drugs (Fig. 1). Many of these chemicals did not have ChEBI IDs. Since many of these chemical elements initially did not exist in ChEBI [32], we requested for 162 new ChEBI additions of these chemical entities. Fig. 1 listed the numbers of ChEBI-recorded chemical components for individual TCDs. For example, Common Threewingnut Root TCD has 47 chemical entities, Tripterygii Hypoglauci Radix TCD has 81 chemical entities, and Common Floweringqince Fruit TCD has the largest number of 125 chemical components (Fig. 1). In total, 555 unique ChEBI IDs were identified from a total of 708 chemical components with ChEBI IDs.

Only 6 TCDs were identified to have various AEs (Fig. 1). These 6 TCDs are associated with 262 AEs, among which 184 are unique. These 6 TCDs and the number of their associated AEs include Common Threewingnut Root TCD (abdominal pain AE, anorexia AE, and gastritis AE), Tripterygii Hypoglauci Radix TCD (nausea and vomiting AE, rash AE, and amenorrhea AE), Nux Vomica TCD, Ovientvine Stem TCD, Debark Peony Root TCD, and Prepared Common Monkshood Branched Root TCD (Fig. 1). In total, 178 AEs were mapped to OAE. We also added to OAE 6 AEs not initially identified in OAE based on the standard OAE development procedures [33].

### OCMR design and development

**Fig. 2:**
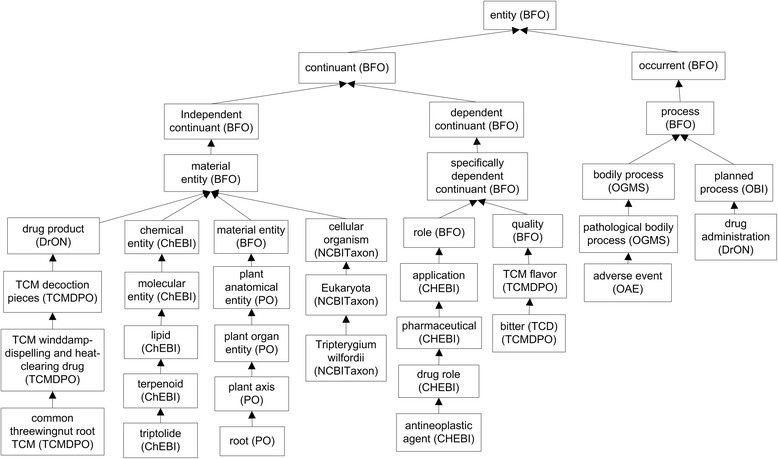
Top level OCMR hierarchy. To make the relations simple and clear, some intermediate terms such as those terms in between Eukaryota and *Triterygium wilfordii* are not shown

Figure 2 shows the basic top-level hierarchical structure of OCMR. Specifically, OCMR uses the Basic Formal Ontology (BFO) [37] as the upper level ontology. BFO contains two branches, continuant and occurrent. The continuant branch represents time-independent entities such as material entity and their quality and roles, and the occurrent branch represents time-related entities such as process and time. Since BFO has been adopted by over 100 biomedical ontologies, the usage of BFO allows OCMR easily and effectively integrated with other ontologies. In OCMR, 555 chemical elements of individual TCDs were mapped to the chemical element terms in the ChEBI ontology. Since higher level terms and relations related to these 555 chemicals were also extracted and included in OCMR, OCMR includes 2194 ChEBI terms. NCBITaxon terms are used to represent organisms. Animal tissues and organs are represented using UBERON, and plant tissues are represented using PO. Since all 26 anti-rheumatism TCDs are decoction pieces, we extracted from the TCMDPO ontology to represent corresponding TCDs.

**Fig. 3:**
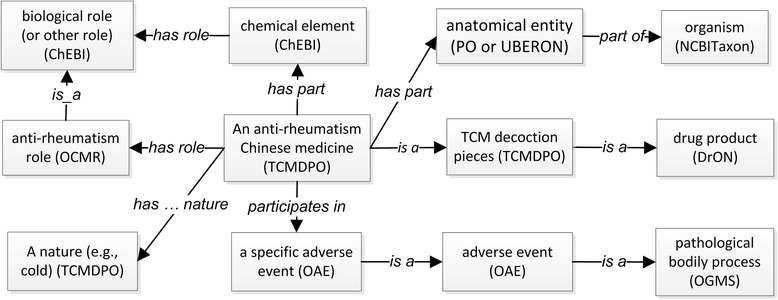
General ontology design pattern for representing the relations among terms in OCMR

**Fig. 4:**
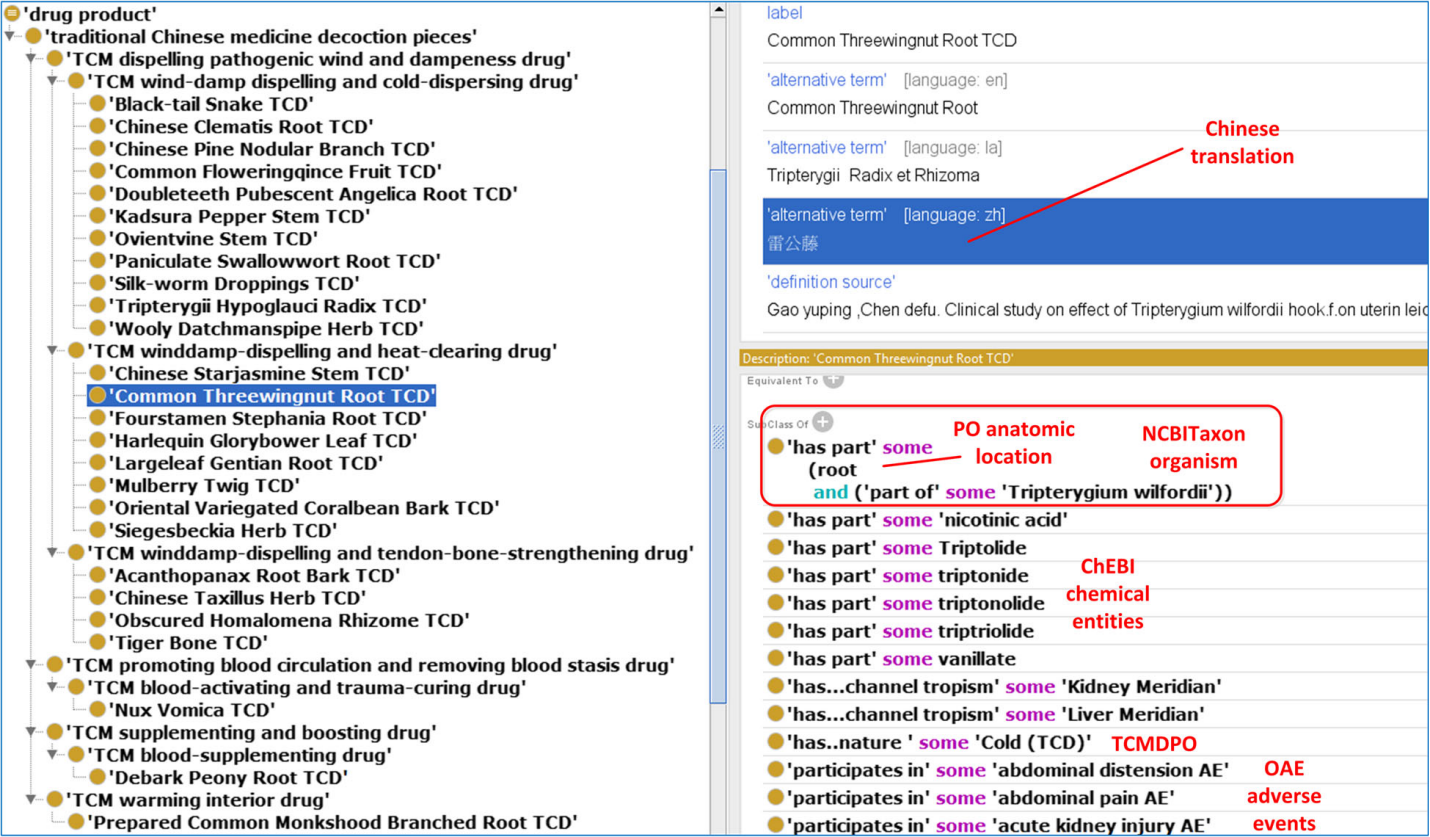
Overview of anti-rheumatism TCDs in OCMR. The left side of the screenshot shows the overview of 26 anti-rheumatism TCDs. The right side of the screenshot shows the annotations and axioms associated with the Common Threewingnut Root TCD. Axioms are generated to represent the relations between this TCD and terms from different ontologies (e.g., NCBITaxon, PO, ChEBI, TCMDPO, and OAE)

Figure 3 provides a general design pattern for a specific anti-rheumatism TCD. Different from hierarchical relations (Fig. 2), this design pattern logically links related terms across different hierarchical structures in OCMR. The semantic relations among different terms are generated using axioms. Figure 4 shows many axioms that link the Common Threewingnut Root TCD to different classes (e.g., chemical entities, AEs) through specific relations. For example, the following axiom defines that the TCD has triptolide as its ingredient part: *‘has part’ some Triptolide*. The relation ‘participates in’ is used to represent a TCD participating in an AE, for example, *Common Threewingnut Root TCD: ‘participates in’ some ‘abdominal distension AE’* (Fig. 4).

The relationships between chemical entities and their biological/application roles, which provide critical information in this study, are represented in ChEBI and OCMR using the object property ‘*has role*’. For example, according to the ChEBI definition, triptolide has a role as an anti-inflammatory agent. ChEBI represents such a relation of triptolide as: *‘has role’ some ‘anti-inflammatory agent’*. In here *‘anti-inflammatory agent’* is classified in ChEBI as an application role. With the relation definitions between TCD, chemical entities, and biological/application roles, we are able to identify important chemical entities with specific roles, which may indicate key mechanisms of anti-rheumatism and AEs.

As described in the Ontobee OCMR statistics page (http://www.ontobee.org/ontostat/OCMR), OCMR contains 3359 class terms, 12 object properties, 116 annotation properties, and 10 instances. As illustrated above, OCMR also generated many logical axioms to link terms imported from different existing ontologies. The additions of these new axioms are based on our manually annotated data (Additional file 1).

In the following sections we will describe our detailed analyses of different OCMR components associated with these anti-rheumatism TCDs.

### Classification and organism sources of 26 anti-rheumatism TCDs

Figure 4 shows the TCMDPO classification of the 26 anti-rheumatism TCDs in OCMR (https://github.com/TCMOntology/TCMDPO). These 26 TCDs are classified into four subclasses. Among them 23 TCDs belong to the subclass of ‘TCM dispelling pathogenic wind and dampness drug’. Interestingly, the two *Tripterygium* species *T. wilfordii* and *T. hypoglaucum* are classified under two different subcategories, that is, *T. wilfordii* TCM is classified as a winddamp-dispelling and heat-clearing drug, and *T. hypoglaucum* as a wind-damp dispelling and cold-dispersing drug (Fig. 4). This observation indicates that two TCDs under the same genus can have different functions (in this case, two *Tripterygium* species drugs but one heat-clearing function and the other cold-dispersing function), suggesting other differences between these two species contribute to the differences. Another interesting observation is that there are three TCD categories each of which only has one TCD (Figs. 1 and 4). Specifically, Nux Vomica TCD, which is made from the seed of *Strychnos nux-vomica*, belongs to the promoting blood circulation and removing blood stasis drug. Debark Peony Root TCD, made from *Paeonia lactiflora Pall*, is classified as a TCM supplementing and boosting drug. Prepared Common Monkshood Branched Root TCD, made from *Aconitum carmichaelii*, belongs to a TCM warming interior drug. All the other 23 TCDs belong to other 3 categories. Such an observation suggests that although the functions of most anti-rheumatism TCDs are grouped under 3 major categories, other anti-rheumatism TCDs have different TCD functions.

**Fig. 5:**
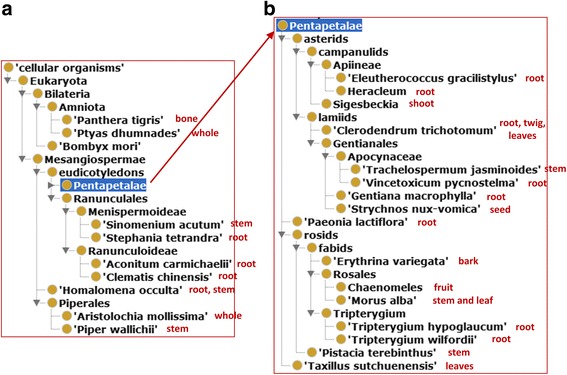
The taxonomy of 26 organisms used for anti-rheumatism TCDs. Ontofox was used to generate the results with the option of “includeComputedIntermediates”. The Protege OWL editor was used for visualization. Among the 26 organisms, 16 belong to the branch of *Pentapetalae*. The anatomic labels next to the organism names represent the anatomic locations from where the Chinese medicines are made from. Root is the most commonly used anatomic location

Our NCBITaxon taxonomy-based analysis found that among the 26 organisms, two are vertebrate animals (i.e., *Amniota*), including tiger (i.e., *Panthera tigris*) and snake (i.e., *Ptyas dhumnades*), and one is silkworm (i.e., *Bombyx mori*) (Fig. 5a). In addition to these 3 *Bilateria* animals, the other 23 organisms belong to *Mesangiospermae* plants (Fig. 5a). Sixteen of these anti-rheumatism TCM plant organisms belong to the subdivision of *Pentapetalae*, including the two *Tripterygium* species *T. wilfordii* and *T. hypoglaucum* (Fig. 5b). This subdivision also includes two *Rosales* species *Chaenomeles* and *Morus alba* (Fig. 5b). It is unclear why the *Pentapetalae* subdivision has so many anti-rheumatism drugs. Note that the Ontofox program is able to identify the closest ancestor for different taxonomy terms [35], such as the *Bilateria* as the closest ancestor for tiger, snake, and silkworm, and *Pentapetalae* as the one for all plant organisms. Overall, our NCBITaxon taxonomy ontology-based study provides more insights in terms of the concrete taxonomical classification of the TCD origins.

### OCMR-based analysis of TCD chemical entities and anti-rheumatism mechanisms

Our OCMR linkage between TCDs, TCD chemical elements, and the hierarchical relations among these chemical elements provides an ideal platform to analyze those chemicals with specific biological roles.

Our analysis has focused on the analysis of the TCD chemical elements that have these two types of roles: anti-inflammatory role and antineoplastic role. By analyzing all chemical elements of these 26 TCDs and their applications/biological or molecular roles, our study identified 18 chemical elements having the anti-inflammatory role and 33 chemical elements having the antineoplastic role (Table 1). Interestingly, we also identified 9 chemical elements that have both anti-inflammatory and antineoplastic roles (Table 1).

**Table 1:** OCMR chemical ingredients having antineoplastic/anti-inflammatory roles

#	Chemical ingredients	ChEBI ID	Chinese Medicine (Chinese name)
Ingredients with both of anti-neoplastic and anti-inflammatory role			
1	4-terpineol	CHEBI_78884	Common Floweringqince Fruit*
2	all-trans-retinoic acid	CHEBI_15367	Silk-worm Droppings, Tiger Bone
3	botulin	CHEBI_3086	Chinese Clematis Root, Common Floweringqince Fruit
4	betulinic acid	CHEBI_3087	Chinese Taxillus Herb, Common Floweringqince Fruit, Debark Peony Root
5	celastrol	CHEBI_63959	Common Threewingnut Root, Tripterygii Hypoglauci Radix
6	ethyl trans-caffeate	CHEBI_132714	Nux Vomica
7	fangchinoline	CHEBI_132893	Fourstamen Stephania Root
8	tripdiolide	CHEBI_9740	Common Threewingnut Root
9	triptolide	CHEBI_9747	Common Threewingnut Root, Tripterygii Hypoglauci Radix
Ingredients with anti-neoplastic role			
10	(+)-sesamin	CHEBI_66470	Acanthopanax Root Bark
11	(+)-syringaresinol	CHEBI_47	Common Threewingnut Root
12	3,4-dihydroxybenzoic acid	CHEBI_36062	Acanthopanax Root Bark, Chinese Taxillus Herb, Common Floweringqince Fruit, Nux Vomica
13	apigenin	CHEBI_18388	Chinese Starjasmine Stem, Harlequin Glorybower Leaf
14	beta-elemene	CHEBI_62854	Wooly Datchmanspipe Herb
15	daidzein	CHEBI_28197	Chinese Clematis Root
16	emodin	CHEBI_42223	Common Threewingnut Root
17	gallic acid	CHEBI_30778	Common Floweringqince Fruit, Nux Vomica
18	genistein	CHEBI_28088	Chinese Clematis Root
19	hesperetin	CHEBI_28230	Chinese Clematis Root
20	hydroxytyrosol	CHEBI_68889	Harlequin Glorybower Leaf
21	isoorientin	CHEBI_17965	Largeleaf Gentian Root
22	luteolin	CHEBI_15864	Common Floweringqince Fruit, Chinese Starjasmine Stem
23	maslinic acid	CHEBI_66682	Common Floweringqince Fruit
24	methoxsalen	CHEBI_18358	Doubleteeth Pubescent Angelica Root
25	morin	CHEBI_75092	Mulberry Twig
26	morusin	CHEBI_7005	Mulberry Ttwig
27	naringin	CHEBI_28819	Chinese Starjasmine Stem
28	neohesperidin	CHEBI_59016	Chinese Clematis Root
29	nobiletin	CHEBI_7602	Chinese Clematis Root, Common Threewingnut Root
30	procyanidin B4	CHEBI_27589	Tripterygii Hypoglauci Radix
31	quercetin	CHEBI_16243	Chinese Clematis Root, Common Floweringqince Fruit, Chinese Taxillus Herb
32	sciscllascilloside E-1	CHEBI_66439	Common Threewingnut Root
33	triptonide	CHEBI_132267	Common Threewingnut Root
Ingredients with anti-inflammatory role			
34	acteoside	CHEBI_132853	Harlequin Glorybower Leaf
35	decanoic acid	CHEBI_30813	Common Floweringqince Fruit
36	ferulic acid	CHEBI_17620	Chinese Taxillus Herb, Nux Vomica
37	lupeol	CHEBI_6570	Chinese Taxillus Herb, Nux Vomica, Silk-worm Droppings
38	maslinic acid	CHEBI_66682	Common Floweringqince Fruit
39	procyanidin B3	CHEBI_75630	Trypterygii Hypoglauci Radix
40	tectorigenin	CHEBI_9429	Chinese Clematis Root
41	triptonide	CHEBI_132267	Common Threewingnut Root
42	fredelin	CHEBI_5171	Tripterygii Hypoglauci Radix

Note: *, for each Chinese drug name (e.g., ‘Common Floweringqince Fruit TCD’), the end “TCD” word is ignored

**Fig. 6:**
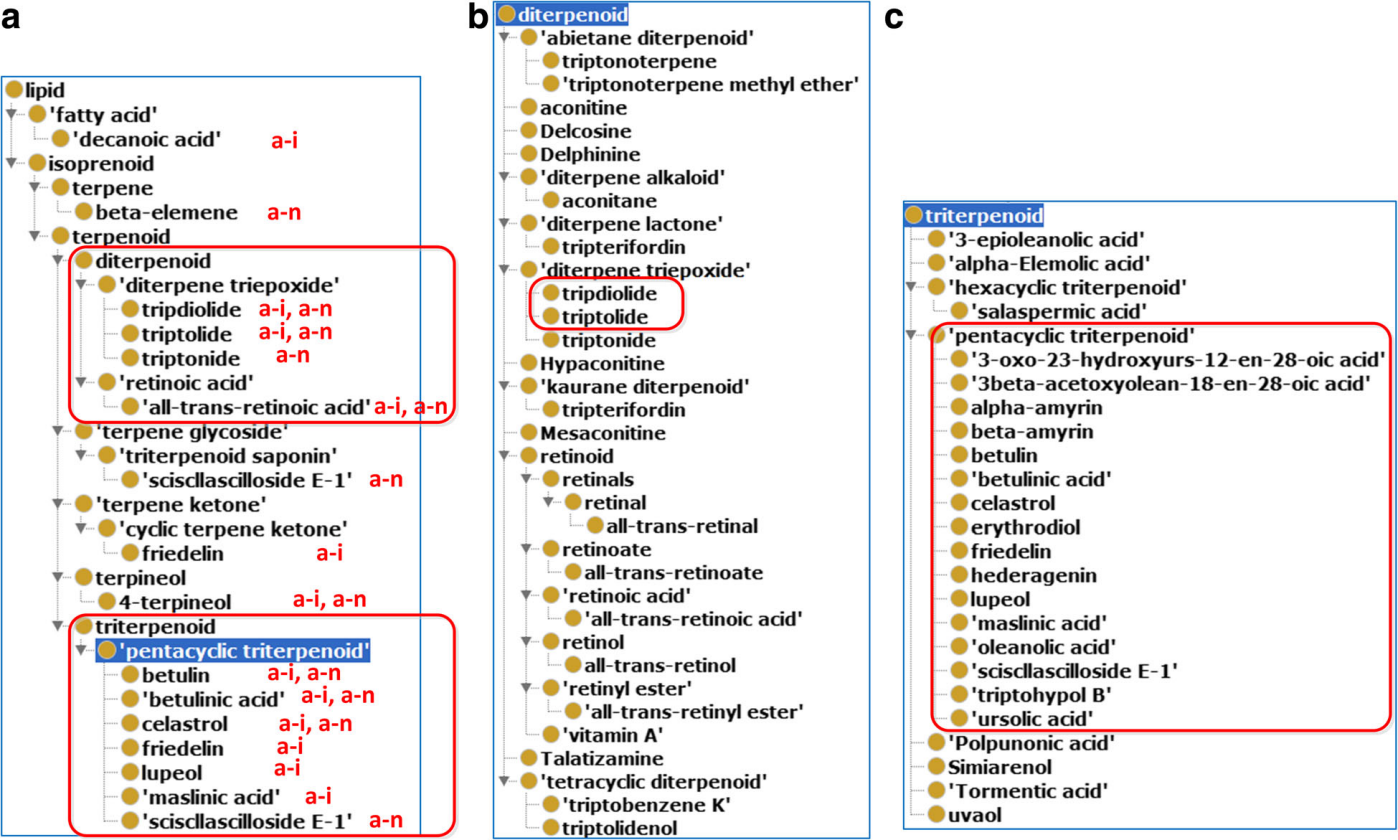
Hierarchical analysis of chemical entities of anti-rheumatism TCDs based on ChEBI classification. **a** 14 chemicals with antineoplastic and/or anti-inflammatory roles are lipids. Among these lipids, 12 are terpenoid chemicals, which include 4 diterpene triepoxides and 7 pentacylic triterpenoids. In the subfigure, “a-i” represents anti-inflammatory role, and “a-n” represents antineoplastic role. To generate this screenshot, Ontofox was used to generate a ChEBI subset using all 42 chemicals with antineoplastic and/or anti-inflammatory roles. The results were visualized using the Protégé OWL editor. **b** The whole OCMR diterpenoid branch that has 22 chemical elements (including triptolide and tripdiolide). **c** The whole triterpenoid branch in OCMR. This branch includes 23 chemical elements, among which are 16 pentacylic triterpenoids

After analyzing the structural classification of the above mentioned 42 chemical entities having anti-inflammatory and/or antineoplastic roles, we identified 14 terpenoids having the anti-inflammatory and/or anti-neoplastic roles (Fig. 6a). The two known bioactive ingredients tripdiolide and triptolide belong to diterpene triepoxide. Among these terpenoids, 4 are diterpenoids (including tripdiolide and triptolide), 7 are triterpenoids (more specifically, pentacyclic triterpenoids). By examining all OCMR listed chemical elements, we further found 22 diterpenoids and 23 triterpenoids (including 16 pentacylic triterpenoids (Fig. 6c). The importance of these findings is described in the Discussion section.

### OCMR-based analysis of anti-rheumatism TCD AEs and possible AE mechanisms

**Fig. 7:**
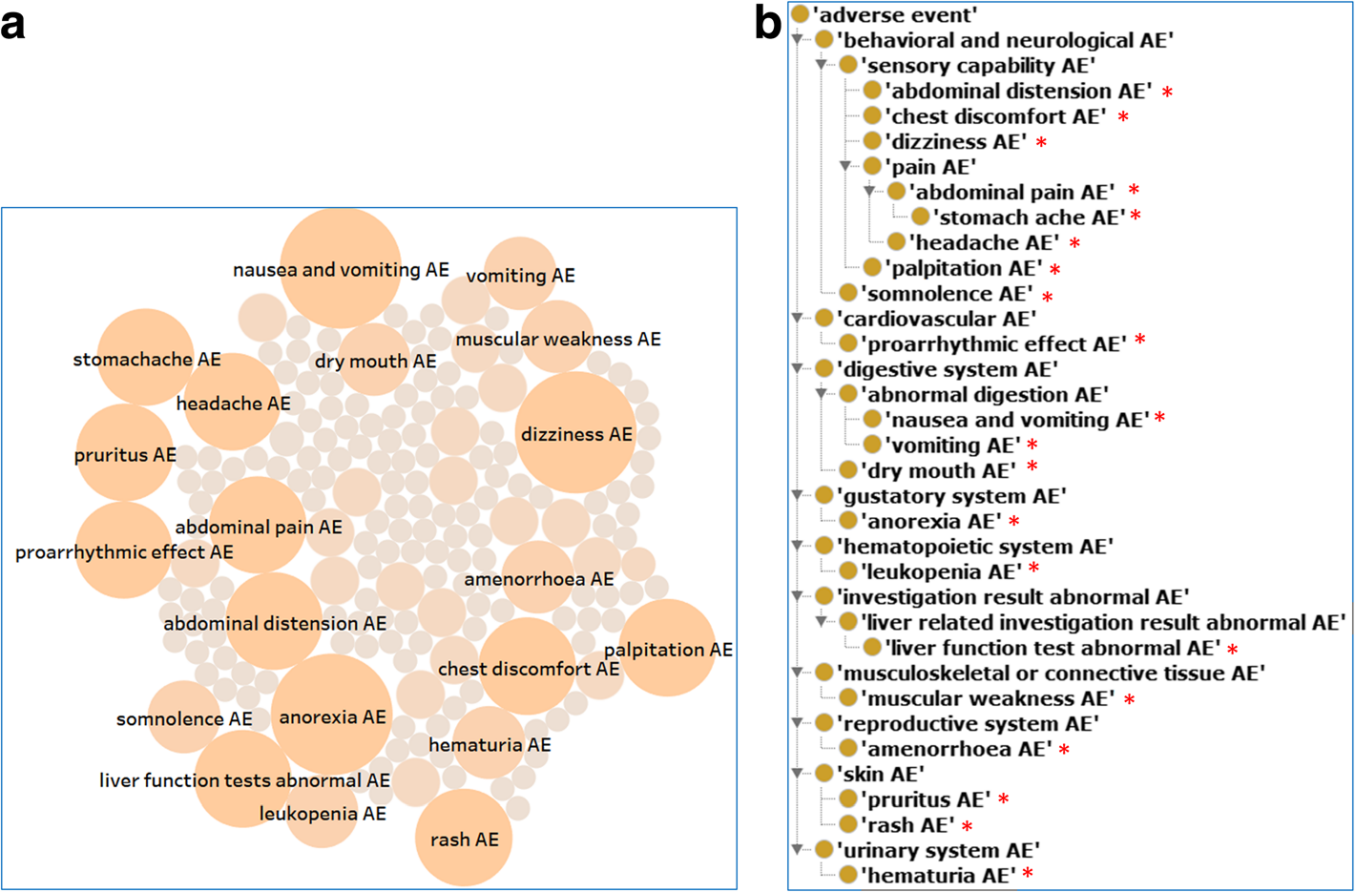
Analysis of AEs associated with anti-rheumatism TCDs. **a** Representation of all 184 unique AEs associated with 6 TCDs. The figure was generated by Tableau. Each circle represents an AE. The size and color of circles represent how many drugs shared the AE. **b** Ontological representation of 20 AEs (indicated by the sign *) each associated with at least 3 TCDs and their associated upper level AE terms. Ontofox was used to generate an OAE subset containing these 20 AEs

The information about AEs provided general safety issues of anti-rheumatism TCDs. In total, 184 unique AEs were identified to be associated with 6 TCDs (Additional file 1). Figure 7a shows an overview of 184 unique AEs associated with 6 TCDs. The top 20 AEs each associated with at least 3 TCDs are labeled. Dizziness, nausea and vomiting, and anorexia are associated with 5 drugs. Ten AEs are associated with 4 drugs. Seven AEs are shared by 3 drugs. To further study these AEs, we utilized the OAE hierarchical structure to classify these 20 AEs into various top-level AE classes in OAE (Fig. 7b). Eight of these 20 AEs (7 of them being sensory capability AEs) belong to the behavioral and neurological AE category. There are 3 digestive system AEs and 2 skin AEs. The remaining 7 AEs belong to different systems (Fig. 7b).

By analyzing the chemical entities and associated biological roles, we can identify possible AE mechanisms. The most prominent side effects of *T. wilfordii* and *T. Hypoglauci* extracts are their association with reduction of reproductive functions including necrospermia, hypomenorrhea, and infertility [16]. Several anti-rheumatism chemical ingredients, including triptolide, tripdiolide, and triptolidenol, have been found to contribute to such antifertility effects [16, 38]. These chemicals have been contained in OCMR. Furthermore, our systematic OCMR knowledge base analysis identified many more neurotoxins and allergens. Interestingly, over 30 benzene type chemicals are found, many of them are known neurotoxins and allergens, such as eugenol (an allergen) [39, 40], diethyl phthalate (neurotoxin with possible adverse reproductive outcomes) [41], and toluene (neurotoxin) [42]. How these benzene chemical entities contribute to known AEs associated with TCDs deserves more studies.

### SPARQL query of the OCMR knowledge base

**Fig. 8:**
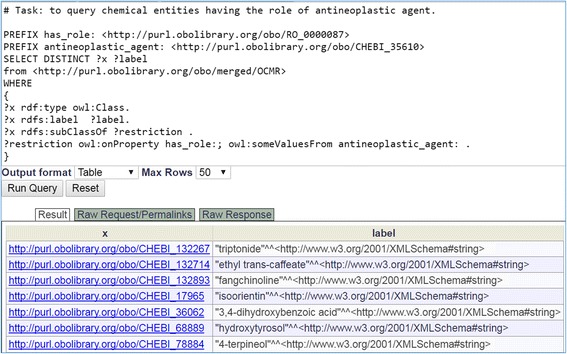
Example SPARQL query of OCMR. Performed on the Ontobee SPARQL website (http://www.ontobee.org/sparql), this example queried all the chemical entities that have the antineoplastic agent role. In total 33 hits were identified, and only 7 are shown in this screenshot

The OCMR ontology is formatted using the Web Ontology Language (OWL) [43] format. The contents of the OWL files can be expressed with Resource Description Framework (RDF; https://www.w3.org/RDF/) triples and stored in an RDF triple store database. The RDF data model makes statements about resources in the form of subject-predicate-object expressions (i.e., triples). SPARQL (a recursive acronym for SPARQL Protocol and RDF Query Language) [44] is used to retrieve data stored in a RDF triple store. Since the OCMR ontology has been stored in the Ontobee triple store, the Ontobee SPARQL website (http://www.ontobee.org/sparql) could be used to query the OCMR knowledge. Figure 8 provides an example of such a SPARQL query. As shown in this example, a few lines of SPARQL code can be used to identify the 33 antineoplastic chemical entities as ingredients of the anti-rheumatism Chinese medicines.

## Discussion

This manuscript reports an interesting bioinformatics project that started simply with the knowledge of a list of anti-rheumatism TCDs. For the list of 26 TCDs, we systematically and manually annotated peer-reviewed articles and retrieved their associated organism sources and anatomic locations in these organisms, chemical ingredients of these Chinese drugs, and AEs. With the collected information, we generated the Ontology of Chinese Medicine for Rheumatism (OCMR). The OCMR systematically represents, organize, and integrates all information related to these anti-rheumatism TCDs. Our OCMR-based analysis was able to generate many scientific insights in terms of the underlying molecular mechanisms for the intended anti-rheumatism and unintended AEs of these TCDs.

One major novelty of our ontology-based approach exists in that OCMR systematically integrates separate information learned from different branches of science in a logical ontology format, supporting advanced data representation, integration, sharing, and analysis. The simple lists of the terms of drugs, chemical elements, and AEs (Additional file 1) are useful but their usage is limited. By mapping these terms to existing ontologies and extracting related information from the existing ontologies (i.e., TCMDPO, NCBITaxon, UBERON, PO, ChEBI, and OAE), we were able to obtain deep background information including their higher layers of class terms in different hierarchical structures and deep relations among these terms. Such extracted information then becomes informative even to people without strong backgrounds in these fields. For example, ChEBI provides additional information about different applications and roles of chemical entities. The collection of total chemicals associated with particular roles (e.g., anti-inflammatory role) allows us to identify all possible chemical entities containing such biological roles. In addition, OCMR includes semantical axioms that logically link different terms in a computer-understandable format. With all terms organized in an integrative OCMR format, we were able to semantically query and analyze the standardized knowledge to address scientific questions.

Our OCMR ontology analysis identified useful insights about anti-rheumatism mechanisms of TCDs. As demonstrated in Table 1 and Fig. 6, our study identified many chemical entities that have anti-inflammatory and/or anti-neoplastic roles. Furthermore, we identified many additional diterpenoids and triterpenoids as chemical components of anti-rheumatism TCDs. Triptolide and tripdiolide are two diterpenoids known to have anti-inflammatory and antineoplastic roles [10–12, 45]. More diterpenoids were identified in our study. Many triterpenoids have also been found to induce anti-inflammatory effects [4, 46–49]. Triterpenoids have been known to be useable as new promising anticancer drugs [50]. Interestingly, the most prominent triterpenoids found in our study are pentacyclic triterpenoids (Fig. 6c). A recent study indicated that pentacyclic triterpenoids inhibit IKKβ-mediated activation of NF-κB pathway [51]. The NF-κB plays an important role in regulating various proinflammatory and immune-regulatory cytokines, cyclo-oxgenase-2, growth factors, and the apoptotic cascade. Given these findings, the potential anti-rheumatism effects of different diterpenoids and triterpenoids (esp. pentacyclic triterpenoids) deserve further informatics and experimental investigations.

The association between anti-rheumatism TCDs and antineoplastic chemical entities is unclear. Our study identified 33 antineoplastic chemical ingredients in anti-rheumatism TCDs. This number nearly doubles the number (18) of anti-inflammatory chemicals detected from our study. Many evidences have been available to associate rheumatism with neoplastia and cancer [22–24]. However, it is still unclear how rheumatic diseases are related to neoplastic conditions [24] and how antineoplastic drugs can help the treatment of rheumatic diseases. Our identification of many antineoplastic chemical ingredients in anti-rheumatism TCDs suggest that these antineoplastic chemical ingredients might play an important role in combinatorial effects against rheumatism.

Unlike western chemical drugs, each TCD usually has many chemicals whose roles in the drug effect are often unknown. Therefore, it is difficult to identify or statistically analyze the chemicals significantly associated with the positive anti-rheumatism effect and the negative AE effects. However, based on the reported roles (e.g., anti-inflammatory and antineoplastic roles) and mechanisms of actions of many chemicals and chemical classifications, we can make general hypotheses as generated and explained in this manuscript. Further conclusions will require experimental verifications in a laboratory animal model(s) with careful controlled randomized study design.

In this study, we only curated, represented, and analyzed traditional Chinese drugs prepared from individual anatomic sources of plant or animal. There are also various combinations of crude drugs used for medication. Such cases are more complicated in terms of its preparation, components, and mechanisms of actions. In addition, it is likely some drugs and drug ingredients interact with each other under specific conditions. Such Chinese drug combinations, their formations and interactions, and the intended and adverse effects of such combinations will be considered in our future ontology-based systematic studies.

Note that after the chemical components of the 26 TCMs are known, it is possible to use ChEBI to do many analyses in terms of chemical classification and mechanism of action study (Table 1, Figs. 5 and 6). Such Ontofox-derived ChEBI subset generation and analysis demonstrate another aspect of ontology usage. It is also realized that without OCMR, the pure ChEBI-based ontological analysis does not allow the direct linkages and queries between chemicals, chemical groups, and TCD drugs, which may diminish integrative applications (e.g., chemical groupings based on more specific TCD drug categories) where such linkages are necessary. In addition to the chemical-based analysis, OCMR provides many more features (e.g., NCBITaxon-based taxonomic analysis) as described in the report. Furthermore, OCMR provides a centralized and reusable knowledge base that can support future revision, extension, storage, and analyses by users with more applications.

The ontology-based methods of anti-rheumatism TCD information representation and analysis described in this study are applicable to other TCDs. Our study demonstrates the usage of the traditional Chinese medicine TCMDPO ontology. TCMDPO has collected 323 class terms and 4 object properties. It contains various data related to TCDs, such as their scientific names, natures, flavors, toxicity and channel tropism. With a small section of the TCMDPO terms and relations imported, OCMR significantly expand the information of these imported TCDs with standardized organisms from NCBITaxon, anatomic entities from PO and UBERON, chemical entities from ChEBI, and AEs from OAE. Furthermore, OCMR logically links different terms with axiom definitions and builds all the information under an integrative BFO-aligned framework. It is possible to extend other TCMDPO drugs using the new strategies implemented in this study. Such an integrative strategy will allow more integrative usage and applications of TCMDPO drugs for more advanced representation, standardization, integration, and analysis of traditional Chinese drugs and their roles in treating various diseases.

## Conclusions

In this study, we first collected 26 anti-rheumatism traditional Chinese drugs and their related information including reported chemical elements and associated adverse event (AEs). Based on the manually annotated data, we developed an Ontology of Chinese Medicine for Rheumatism (OCMR). OCMR has three main features: (i) By importing related terms from existing ontologies, OCMR represents hierarchical structures of these traditional Chinese drugs, their associated organisms, anatomic locations, chemical entities and their roles, and AEs. (ii) OCMR uses semantic axioms to link terms from different hierarchical structures (e.g., drugs and chemical entities). (iii) OCMR integrates all the terms and their relations in a BFO-aligned systemic framework. Our OCMR ontology analysis identified many chemical entities with anti-inflammatory and antineoplastic roles, which may explain drug anti-rheumatism mechanisms, and we also identified chemicals with neurotoxin or allergen roles, which many explain drug AEs. Important branches of chemical entities, such as diterpenoids and triterpenoids, were also identified. Based on these findings, we also propose new areas of future anti-rheumatism research. This study represents the first study of using ontology to systematically represent and analyze traditional Chinese drugs, their chemical ingredients and associated roles, and AEs. Such a method can also be applied to study other traditional Chinese drugs.

## Additional file

Additional file 1: An Excel file of all the detailed information. This Excel file includes four spreadsheets. These spreadsheets cover the following information:Spreadsheet 1: TCD-related organisms, organism IDs in NCBITaxon. Spreadsheet 2: Chemical components in TCDs, ChEBI IDs, and related references. Spreadsheet 3: TCD AEs including AE names, OAE AE IDs, related references. Spreadsheet 4: TCD-specific anatomic locations in organisms, and the PO and UBERON IDs corresponding to these anatomic entities. (XLSX 93 kb)

## Abbreviations

AE: Adverse event
BFO: Basic Formal Ontology
ChEBI: Chemical Entities of Biological Interest
DrON: Drug Ontology
NCBI: National Center for Biotechnology Information
NCBITaxon: NCBI Taxonomy Ontology
OAE: Ontology of Adverse Events
OBO: Open Biological/Biomedical Ontologies
OCMR: Ontology of Chinese Medicine against Rheumatism
OGMS: Ontology for General Medical Science
OWL2: Web Ontology Language 2
PO: Plant Ontology
RDF: Resource Description Framework
SPARQL: SPARQL Protocol and RDF Query Language
TCD: Traditional Chinese Drug
TCM: Traditional Chinese Medicine
TCMDPO: TCM Decoction Pieces Ontology

## References

[1] Parish LC (1963). An historical approach to the nomenclature of rheumatoid arthritis. Arthritis Rheum.

[2] Joshi VR, Poojary VB (2013). William Heberden (elder) (1710-1801). J Assoc Physicians India.

[3] Sangha O (2000). Epidemiology of rheumatic diseases. Rheumatology.

[4] Yang CL, Or TC, Ho MH, Lau AS (2013). Scientific basis of botanical medicine as alternative remedies for rheumatoid arthritis. Clin Rev Allergy Immunol.

[5] Gossec L, Smolen JS, Ramiro S, de Wit M, Cutolo M, Dougados M (2016). European league against rheumatism (EULAR) recommendations for the management of psoriatic arthritis with pharmacological therapies: 2015 update. Ann Rheum Dis.

[6] Chen CY, Tsai CY (2014). From endocrine to rheumatism: do gut hormones play roles in rheumatoid arthritis?. Rheumatology.

[7] Liu Y, Tu S, Gao W, Wang Y, Liu P, Hu Y (2013). Extracts of Tripterygium wilfordii hook F in the treatment of rheumatoid arthritis: a systemic review and meta-analysis of randomised controlled trials. Evid Based Complement Alternat Med.

[8] Tao X, Lipsky PE (2000). The Chinese anti-inflammatory and immunosuppressive herbal remedy Tripterygium wilfordii hook F. Rheum Dis Clin N Am.

[9] Tao X, Younger J, Fan FZ, Wang B, Lipsky PE (2002). Benefit of an extract of Tripterygium Wilfordii hook F in patients with rheumatoid arthritis: a double-blind, placebo-controlled study. Arthritis Rheum.

[10] Tao X, Fan F, Hoffmann V, Gao CY, Longo NS, Zerfas P (2008). Effective therapy for nephritis in (NZB x NZW)F1 mice with triptolide and tripdiolide, the principal active components of the Chinese herbal remedy Tripterygium wilfordii hook F. Arthritis Rheum.

[11] Ma J, Dey M, Yang H, Poulev A, Pouleva R, Dorn R (2007). Anti-inflammatory and immunosuppressive compounds from Tripterygium wilfordii. Phytochemistry.

[12] Tao X, Cai JJ, Lipsky PE (1995). The identity of immunosuppressive components of the ethyl acetate extract and chloroform methanol extract (T2) of Tripterygium wilfordii hook. F. J Pharmacol Exp Ther.

[13] Wang T, Shen F, Su S, Bai Y, Guo S, Yan H (2016). Comparative analysis of four terpenoids in root and cortex of Tripterygium wilfordii radix by different drying methods. BMC Complement Altern Med.

[14] Ho LJ, Lai JH (2004). Chinese herbs as immunomodulators and potential disease-modifying antirheumatic drugs in autoimmune disorders. Curr Drug Metab.

[15] Zhong J, Xian D, Xu Y, Liu J (2011). Efficacy of Tripterygium hypoglaucum hutch in adults with chronic urticaria. J Altern Complement Med.

[16] Li XJ, Jiang ZZ, Zhang LY (2014). Triptolide: progress on research in pharmacodynamics and toxicology. J Ethnopharmacol.

[17] Zheng Q, Zhao Y, Wang J, Liu T, Zhang B, Gong M (2014). Spectrum-effect relationships between UPLC fingerprints and bioactivities of crude secondary roots of Aconitum Carmichaelii Debeaux (Fuzi) and its three processed products on mitochondrial growth coupled with canonical correlation analysis. J Ethnopharmacol.

[18] Chan TY, Critchley JA (1996). Usage and adverse effects of Chinese herbal medicines. Hum Exp Toxicol.

[19] Conn DL (2001). Resolved: low-dose prednisone is indicated as a standard treatment in patients with rheumatoid arthritis. Arthritis Rheum.

[20] Misischia RJ, Moreland LW (2002). Rheumatoid arthritis: developing pharmacological therapies. Expert Opin Investig Drugs.

[21] Rao P, Knaus EE (2008). Evolution of nonsteroidal anti-inflammatory drugs (NSAIDs): cyclooxygenase (COX) inhibition and beyond. J Pharm Pharm Sci.

[22] Abu-Shakra M, Buskila D, Ehrenfeld M, Conrad K, Shoenfeld Y (2001). Cancer and autoimmunity: autoimmune and rheumatic features in patients with malignancies. Ann Rheum Dis.

[23] Benedek TG (1988). Neoplastic associations of rheumatic diseases and rheumatic manifestations of cancer. Clin Geriatr Med.

[24] Bojinca V, Janta I (2012). Rheumatic diseases and malignancies. Maedica.

[25] Blake JA, Bult CJ (2006). Beyond the data deluge: data integration and bio-ontologies. J Biomed Inform.

[26] Hoehndorf R, Schofield PN, Gkoutos GV. The role of ontologies in biological and biomedical research: a functional perspective. Brief Bioinform. 2015;16(6):1069–80. https://www.ncbi.nlm.nih.gov/pubmed/25863278. 10.1093/bib/bbv011 PMC4652617 25863278

[27] Bodenreider O. Biomedical ontologies in action: role in knowledge management, data integration and decision support. Yearb Med Inform. 2008:67–79. PMC2592252 18660879

[28] Schulz S, Balkanyi L, Cornet R, Bodenreider O (2013). From concept representations to ontologies: a paradigm shift in health informatics?. Healthc Inform Res.

[29] NCBITaxon: An ontology representation of the NCBI organismal taxonomy. http://obofoundry.org/ontology/ncbitaxon.html. Accessed on July 29, 2017.

[30] Cooper L, Walls RL, Elser J, Gandolfo MA, Stevenson DW, Smith B (2013). The plant ontology as a tool for comparative plant anatomy and genomic analyses. Plant Cell Physiol.

[31] Mungall CJ, Torniai C, Gkoutos GV, Lewis SE, Haendel MA (2012). Uberon, an integrative multi-species anatomy ontology. Genome Biol.

[32] Hastings J, de Matos P, Dekker A, Ennis M, Harsha B, Kale N (2013). The ChEBI reference database and ontology for biologically relevant chemistry: enhancements for 2013. Nucleic Acids Res.

[33] He Y, Sarntivijai S, Lin Y, Xiang Z, Guo A, Zhang S (2014). OAE: the ontology of adverse events. J Biomed Semantics.

[34] Smith B, Ashburner M, Rosse C, Bard J, Bug W, Ceusters W (2007). The OBO foundry: coordinated evolution of ontologies to support biomedical data integration. Nat Biotechnol.

[35] Xiang Z, Courtot M, Brinkman RR, Ruttenberg A, He Y (2010). OntoFox: web-based support for ontology reuse. BMC research notes.

[36] Xiang Z, Zheng J, Lin Y, He Y (2015). Ontorat: automatic generation of new ontology terms, an-notations, and axioms based on ontology design patterns. J Biomed Semantics.

[37] Grenon P, Grenon P (2003). Spatio-temporality in Basic Formal Ontology. IFOMIS reports.

[38] Zhen QS, Ye X, Wei ZJ (1995). Recent progress in research on Tripterygium: a male antifertility plant. Contraception.

[39] Lopez-Saez MP, Carrillo P, Huertas AJ, Fernandez-Nieto M, Lopez JD (2015). Occupational asthma and dermatitis induced by eugenol in a cleaner. J Investig Allergol Clin Immunol.

[40] Silvestre JF, Albares MP, Blanes M, Pascual JC, Pastor N (2005). Allergic contact gingivitis due to eugenol present in a restorative dental material. Contact Dermatitis.

[41] Swan SH (2008). Environmental phthalate exposure in relation to reproductive outcomes and other health endpoints in humans. Environ Res.

[42] Filley CM, Halliday W (2004). Kleinschmidt-DeMasters BK. The effects of toluene on the central nervous system. J Neuropathol Exp Neurol.

[43] W3C. OWL 2 Web Ontology Language Quick Reference Guide (Second Edition), W3C Recommendation 11 December 2012. 2012: http://www.w3.org/TR/owl2-quick-reference/. Accessed on December 10, 2016.

[44] Harris S, Seaborne A. SPARQL 1.1 Query Language, W3C Recommendation 21 March 2013. 2013: URL: http://www.w3.org/TR/sparql11-query/, accessed on December 26, 2016.

[45] Leuenroth SJ, Crews CM (2005). Studies on calcium dependence reveal multiple modes of action for triptolide. Chem Biol.

[46] de Almeida BC, Araujo BQ, Carvalho AA, Freitas SD, Maciel DD, Ferreira AJ (2016). Antiprotozoal activity of extracts and isolated triterpenoids of 'carnauba' (Copernicia Prunifera) wax from Brazil. Pharm Biol.

[47] Choi SP, Choi CY, Park K, Kim N, Moon HS, Lee D (2016). Glabretal-type triterpenoid from the root bark of Dictamnus dasycarpus ameliorates collagen-induced arthritis by inhibiting Erk-dependent lymphocyte proliferation. J Ethnopharmacol.

[48] Jingbo W, Aimin C, Qi W, Xin L, Huaining L (2015). Betulinic acid inhibits IL-1beta-induced inflammation by activating PPAR-gamma in human osteoarthritis chondrocytes. Int Immunopharmacol.

[49] Baek SY, Lee J, Lee DG, Park MK, Lee J, Kwok SK (2014). Ursolic acid ameliorates autoimmune arthritis via suppression of Th17 and B cell differentiation. Acta Pharmacol Sin.

[50] Petronelli A, Pannitteri G, Testa U (2009). Triterpenoids as new promising anticancer drugs. Anti-Cancer Drugs.

[51] Patil KR, Mohapatra P, Patel HM, Goyal SN, Ojha S, Kundu CN (2015). Pentacyclic Triterpenoids inhibit IKKbeta mediated activation of NF-kappaB pathway: in Silico and in vitro evidences. PLoS One.

